# Perspectives from parents and clinicians on an ecology-focused approach to a group well-child care

**DOI:** 10.1186/s12875-025-02718-z

**Published:** 2025-02-01

**Authors:** Ishaan T. Shah, Nina D. Ham, Hassan Lubega, Christopher L. Boswell, Esther Kim Liu, Renée D. Boynton-Jarrett

**Affiliations:** 1 Department of Pediatrics, Boston University School of Medicine, Boston, MA USA; 2 Department of Family Medicine, Mayo Clinic, Rochester, MN USA; 3 University of California Davis School of Medicine, Davis, CA USA; 4 Department of Pediatrics, University of Maryland Baltimore Washington Medical Center, Baltimore, MD USA

**Keywords:** Nature, Ecology, Pediatrics, Group well-child care, Prevention, Counseling

## Abstract

**Background:**

Group well-child care (GWCC) is a novel group-based alternative for pediatric primary care visits that may allow for adaptations that better tailor to the needs of underserved populations. This qualitative study investigates clinician and parent perspectives on the acceptability and feasibility of integrating ecology-focused content in GWCC using semi-structured interviews with GWCC parent-graduates and ecology-focused child clinicians.

**Methods:**

Ecology-focused child clinicians were purposively sampled via email outreach. GWCC parent graduates were recruited via announcement in private Facebook groups. One-on-one interviews were conducted via videoconference, transcribed, and analyzed using an inductive approach. Parent and clinician thematic analyses were independently conducted to construct shared domains.

**Results:**

Nine GWCC parent-graduates and nine ecology-focused child clinicians were recruited into the study. Four overarching themes were constructed across parent and clinician responses: questions about clinical appropriateness, parent and clinician desires for educational support, influences of perceptions of nature on clinicians, and parent desires to develop independence and autonomy.

**Conclusion:**

This study identified nuanced considerations from the perspective of parents and clinicians for the implementation of ecology-focused content in the GWCC setting. Understanding the range of preferences parents and clinicians may have over ecology-focused content can help GWCC clinicians in designing ecology-focused preventive counseling materials.

**Supplementary Information:**

The online version contains supplementary material available at 10.1186/s12875-025-02718-z.

## Background

Interventions which explore nature with children and families have expanded into educational and healthcare settings. A growing body of research documents the benefits of contact with nature for children and families, such as improved mental health [1], birth outcomes [2], obesity management [3], socioemotional function and development, and physical activity [4]. In educational settings, teachers have suggested that school garden curriculums provide rich sensory experiences that support cognitive development [5, 6]. In the healthcare setting, clinical trials discuss how medical offices that promote nature contact, referred to as “park prescriptions,” impact patients [1, 7, 8]. We see these diverse adaptations to school and health-care delivery as under a common approach we have defined as “ecology-focused.” Briefly, this term is defined as an approach which centers the cultural relevance of the human-nature interaction and the various interrelationships which it encompasses. Creative modalities to incorporate ecology-focused interventions into educational and healthcare settings continue to be of interest to clinicians.

Group well-child care (GWCC) may allow for further refinement of ecology-focused interventions in the pediatric setting. GWCC, CenteringParenting® being one common model, brings together a small group of parents with similarly aged children for well-child visits with developmentally appropriate individual screenings, vaccination, and assessment by a clinician coordinated with facilitated group discussion [9, 10]. Emerging research, including a randomized trial of GWCC, has demonstrated its effectiveness in enhancing vaccination timeliness, appointment attendance, and overall satisfaction with care [11–13]. Evidence suggests that children in lower-income families receive insufficient developmental and behavioral screenings and preventive services and that GWCC models may address these limitations, increase access to health services, and provide opportunities for social connection [14–16]. The flexibility in the delivery of GWCC allows it to be tailored to unique populations and communities [17–19]. Adaptations to GWCC have previously involved modifications to teach positive parenting, encourage home safety, and promote primary obesity prevention [20–22].

Ecology-focused clinicians are striving to integrate novel interventions into pediatric clinical practice [1, 4, 7]. GWCC offers an innovative approach to preventive care that has the potential to incorporate diverse content. Concurrently, a seasoned cohort of parent-graduates of GWCC models and a passionate community of expert pediatric GWCC clinicians have been demonstrating the scalability of the model [12, 20, 21, 23]. To date, the potential synergy between these models remains unexplored. This is the first study to investigate both clinician and parent perspectives on the acceptability and feasibility of integrating ecology-focused content into GWCC. These findings fill a critical gap in the literature by exploring diverse stakeholder perspectives and strategies to improve the integration of holistic care within GWCC settings.

## Methods

### Study design

Interview questions were developed through formative pilot research engaging parent and clinician community partners of Vital Village Networks, a Boston-based community engagement network, in a series of scoping interviews. These interview guides were piloted with a parent-advisor who had experience with GWCC, as well as with clinicians familiar with GWCC (CB).

Initial engagement revealed a broad range of topics that parent and clinician partners believed were at the intersection of nature and preventive pediatric care. Consequently, the scope of the discussion was defined by the term “ecology-focused.” This term was used to describe experiences/concepts where human-nature interaction and its interconnected relationships was perceived to be important to parents and pediatric clinicians due to social and cultural relevance.

For clinician interviews, a literature review (methodologic details provided in Appendix D) was conducted to more fully characterize the domains where ecology-focused material demonstrated relevance to pediatric anticipatory guidance as identified in the American Academy of Pediatrics Bright Futures guidelines [24–27]. Seven domains arose from this iterative process. The goal of the study was to seek out clinicians that as a group had expertise that encompassed these domains. The parent experience in GWCC adaptations was also of interest. Parents with experiences in GWCC are a unique population who may have additional expertise on clinical care redesign due to their firsthand participation in a unique care model [14, 28–30]. Previous research on GWCC and group prenatal care (GPNC) has utilized parent expert advisory committees of previous participants to shape curricular design [20, 31, 32].

### Recruitment

This study protocol was approved by the Institutional Review Board (IRB) of Boston University Medical Center. In this qualitative study, we aimed to explore perspectives of clinicians and parents on the integration of ecology-focused components into GWCC. Study recruitment and interviews took place between June 2022 and September 2023. Ecology-focused child clinicians were purposively sampled based on expertise at the intersection of nature and pediatrics and recruited via email. For parent participants, inclusion criteria required participation in GWCC with a previous child. GWCC parent-graduates were recruited through social media announcements that were co-designed by a parent and clinician champion (EL) who has experience with GWCC. Recruitment announcements were shared by a parent and clinical champion to internal Facebook groups with parents who have previously participated in GWCC in Maryland and Boston. Semi-structured interviews, conducted virtually using videoconferencing software, were administered by trained researchers (NH, ITS) and participants were provided with a $40 gift card upon completion.

A total of 24 ecology-focused child clinicians were identified through an online search of publicly-facing academic profiles. Of the 24 clinicians invited, 9 were interviewed between September 2022 and July 2023. A total of 15 GWCC parent-graduates contacted the research team after seeing a social media post in their GWCC Facebook group. Of those who initially responded, nine were interviewed between June 2022 and September 2023. Interviews lasted approximately 33–57 (average = 45.2) minutes for parents and 35–61 (average = 50.6) minutes for clinicians.

### Parent semi-structured interview protocol

The semi-structured interview protocol for GWCC parent-graduates consisted of three parts: asking about (1) experiences with children and families in nature, (2) intersections between pediatric healthcare and nature, and (3) the feasibility and acceptability of incorporating ecology-focused themes into GWCC. Each section took approximately 20 min to complete.

### Clinician semi-structured interview protocol

Each semi-structured interview with an ecology-focused child clinician consisted of three distinct 20-minute sections. First, the interview protocol invited clinicians to select one of the following seven domains: Socio-Emotional Wellbeing; Built Environment and Environmental Toxins; Play and Learning; Nutrition and Diet; Microbes; Language and Reading; and Technology. Second, clinicians in the study were invited to discuss professional familiarity with GWCC practices and approaches. Finally, the interview evaluated feasibility and acceptability of integrating domain-specific activities into the framework of GWCC. This structured approach allowed for comprehensive exploration of both the clinicians’ expertise and their perspectives on the potential integration of ecology-focused activities into the GWCC model. [Interview guides for parents and clinicians are available in Appendix E].

### Analysis

Two parallel coding and thematic analysis processes were used for the clinician and parent interviews. For both samples, interviews were recorded, transcribed, and uploaded to Dedoose software (version 9.0, SocioCultural Research Consultants, LLC, Los Angeles, CA) for analysis. Three investigators (ITS, NH, RBJ) collaborated to develop a unique and comprehensive code dictionary for each sample [33]. Each transcript was independently coded using an inductive thematic approach [34]. Following individual coding, investigators (ITS, NH, RBJ) reviewed coding outcomes and reconciled disparities to achieve consensus, thus facilitating identification of themes through iterative analysis. This process ensured a rigorous exploration of the data and the identification of nuanced themes. Following independent thematic analysis processes for clinician and parent interviews, consensus themes were categorized into overarching domains.

## Results

Three parent and four clinician themes were constructed from the iterative process. From these themes, four domains were synthesized across parent and clinician interviews. These four domains were: (1) appropriateness of ecology-focused interventions in the GWCC clinical setting, (2) educational supports desired by parents and clinicians to conduct and participate in group visits, (3) clinician commentary on perceptions and understanding of nature, and (4) parents’ desire for independence and exploration. Overarching domains and their intersection with parent and clinician themes are highlighted in Table 1.

**Table 1:** Core domains and parent and clinician themes

Domain	Parent Themes	Clinician Themes
Clinical Appropriateness	Parents largely feel that the incorporation of ecology-focused activities into GWCC would be a well received component of clinical care.	Clinicians recommended a personalized approach to implementation that can adapt to include groups of families from differing backgrounds. Clinicians’ suggestions about ecology-focused GWCC activities are often connected to diverse aspects of pediatric anticipatory guidance in age, timing, and topic.
Educational Support	Parents expressed a desire for their health care professionals to provide more guidance and education on child safety and assessing risk when interacting with nature.	Clinicians want to leverage local resources and knowledge to integrate community characteristics and historical expertise into the ecology-focused activity design in GWCC.
Cultural Understandings of Nature		Clinicians observed variation in perceptions of nature among families and themselves based on personal experiences and cultural contexts.
Independence & Exploration	Parents perceive nature as a valuable experience for themselves and their children to build independence and to explore their surroundings.	

### Clinical appropriateness

#### Clinicians

Clinicians in the study have some hesitations about incorporating ecology-focused GWCC activities. For instance, they are worried about cultural and socioeconomic disparities in nature experiences and challenges to inclusivity in an ecology-focused GWCC visit.

“*Communities of color*,* lower socioeconomic status communities*,* non-English language speaking communities. All*,* I think*,* there’s a lot of barriers in the US at least to participating in those sorts of things. Even if you think about 'Oh*,* there’s a county park with trails. Who’s allowed to go on those trails? What’s the rules or the etiquette of being on those trails? Think about paddling*,* right? Am I allowed to just launch a boat from anywhere? Will I get into trouble? Am I supposed to be out on the water here?' If you don’t have someone kind of guiding you into that*,* how would you know?*” (Clinician).

Several clinicians in the study acknowledge that parents struggle with balancing the desire to have their children explore the outdoors against feelings of danger and uncertainty that are linked to nature spaces their family has access to.

“*But I think it’s not enough*,* because I think people will not go if there’s not programming. If they don’t feel safe there*,* or if they’re experiencing other barriers that haven’t been addressed. It might just feel like ‘here’s another thing they’re telling me to do.’ But if my barrier is that ‘I don’t feel safe. I don’t come home till 10 PM. I’m not with my child during daytime hours or we don’t like to go outside when it’s cold and rainy*,*’ and you live in a place where it’s cold and rainy like 9 months out of the year.*” (Clinician).

#### Parents

While parents also acknowledged concerns with inclusivity in WCC design, they advocated for continued creative iterations of GWCC that incorporated ecology concepts. Parents expressed that challenges due to differences in cultural and socioeconomic backgrounds already affect traditional WCC and may also affect GWCC, particularly in accessing medical care.

“*I think it just harks back on to like*,* there’s a different sense of*,* of being like when you’re crammed into an office or that sterile environment*,* like it’s very*,* some people have like that white coat mindset where you’re anxious or you feel uncomfortable where I know for my family*,* when we walk outside*,* it’s a different sense. We all feel differently. And I would love to experience that with that group. So it’s a more positive experience because these are well-baby*,* happy-baby checkups. It’s not nobody’s sick*,* nobody’s hurt. Like we should be able to change our location.*” (Parent).

Parents have specific concerns they would want addressed in an ecology-focused GWCC setting. They are looking for risk and safety guidance about life experiences at the intersection of nature and child health. This can include what age to introduce certain foods that are perceived as natural or organic, what activities in nature are safe at particular ages, and a clinician safety assessment of homeopathic versus allopathic treatment options.

“*Yeah*,* so I wanna hear from my providers. I do like to listen to other people to say like these are the recommendations. I of course do a lot of research on my own*,* but hearing it from my own provider will let me know like this is accepted. So like even if it’s just throw a blanket down on some grass and put your baby on it*,* or like it’s okay if your baby eats a blade of grass. Like I want reassurance doing these activities and not stress about them before they happen.*” (Parent).

### Educational Support

#### Clinicians

Clinicians would like more coordination and professional support incorporated into GWCC activities. Many clinicians expressed interest in using an outside expert with a different educational background as a resource for ecology-focused GWCC.

“*I would probably try to bring in other experts too just knowing how I like to teach. For example*,* if I am going to talk about 4-month olds and we know that we have that time*,* and we know that the 4-month olds are more stimulated and waking up at night*,* I would bring in an expert whether it’s virtual or in-person to talk about sleep and supporting healthy sleep and sleep teaching. A lot of parents are going to start getting interested in that sleep teaching between 4 and 6 months of age.”* (Clinician).

Many clinicians in the study were also interested in connecting different resources together and aligning curricula across multiple societal sectors (education, healthcare, environmental protection, etc.) that work closely with children and families.

“*I think all of these models. the direction where we do need to go is thinking less about them in isolation*,* and thinking about how we stack them*,* and how are we finding the synergy across these different things*,* whether it’s nature*,* whether it’s reading*,* whether it’s the way we’re delivering well-child care whether it’s the way we’re connecting with schools and programs that are happening there. The extent that we can stack is most important.”* (Clinician).

#### Parents

For GWCC parent-graduates, GWCC already plays an important role for risk management and discussions around safe activities for the family. [For a full documentation of GWCC-related insights from parents including novel facilitation suggestions, topic timing, and group space redesign among other subjects, please refer to Appendix B.] For parents, GWCC can be a platform through which varied discussions on risk assessment of nature experiences can happen.

“*Not right now. But again*,* like*,* as she gets older*,* how do you introduce … at what point do you tell your kid to stop rolling around in dirt and putting it in their mouth? You know*,* and I’m sure that as she gets older*,* those conversations are going to kind of become more natural. But at this point in time*,* every concern that I’ve had has been a discussion in our CenteringParenting® group that has kind of helped ease my fears as far as exposing my child to them or things of that nature.*” (Parent).

Parents want reassuring evidence around their choices to interact with activities and environments in nature. They would also appreciate suggestions around appropriate ecology-focused activities at different ages.

“*I really want to just hear about their experience and their expertise in*,* I’m trying to think of the best way to work this*,* in exposing my child to different things. We talk a lot about*,* you know*,* as our kids are growing up*,* we talk a lot about the different foods that they eat*,* right? And the things like peanut allergies is a big thing so this is how you should approach it. So I’m kind of hoping that same thing with like nature*,* that there’s going to be some way that they’re able to say*,* okay*,* yes*,* the thought of having my kid having a peanut allergy is scary*,* but if you do this and we expose them in this way*,* then we can be sure that it’s not going to be an issue. And I’m hoping that they’re going to be able to guide me with different things with outdoor activities or things of that nature*,* as they would for things like nutrition.”* (Parent).

### Cultural understandings of Nature

Clinicians in the study often talked about nature in ways that coincided with their professional background and their own experiences with nature. Clinicians’ opinions about nature and health were sometimes influenced by their perceptions of societal changes around them.

“*But unfortunately*,* parents raising kids now have to make so many decisions that are environmentally related unfortunately with climate change*,* it’s going to just get worse. How these things play a role in higher temperatures*,* and with the more polluted air*,* higher temperatures. These things tend to concentrate at the microenvironmental area of kids that are closer to the ground. It’s going to be even more complicate*d.” (Clinician).

Clinicians sometimes explained how nature impacts human health on a macro-to-micro scale and brought in considerations of temporal relationships between humans and nature. Clinicians repeatedly demonstrated an internal locus of what nature and ecology means to them that is shaped by complex factors.

“*The understanding of creation stories allow an individual to place themselves and their families*,* their extended families*,* their communities within this context so that it’s not just an isolated living experiment. That is*,* it’s an understanding of connectiveness and it is the relationship that one has with the past as well as the relationship one has with the future […] So I think when we think about how we define nature*,* it really can be broader even that there’s that micro and macro opportunity to gain understanding.*” (Clinician).

Clinicians in the study observed these variations in perceptions of nature not only in themselves but also in their patients. [A more comprehensive selection of quotes discussing perceptions of nature by parents and clinicians which includes comments about temporal and geographic variation in nature experiences, responsibilities of humans to care for the natural world, and ideas about what constitutes natural versus unnatural among other concepts is available in Appendix C]. They felt that a variety of professional and personal approaches of building human-nature connection was needed to engage families. In addition, they suggested that GWCC had to adapt to cultural and community contexts, even adjusting to the identity of the clinicians facilitating the visits.

“*I think that that’s a question that is really important*,* that it is unique and individualized for different families and different locations. It depends where you live. It depends culturally what you enjoy doing because different people like doing different things outside. And not everybody loves being outside. Sometimes it’s a process*,* it’s an education. And if we really want them to be outside*,* it’s a learning curve and a learning experience for children and families*.” (Clinician).

### Independence & Exploration

Parents perceive nature as a valuable experience for children to explore their surroundings. Many parents acknowledge the personal growth and satisfaction they gain from watching their children explore the world during family nature-related engagements.

“*My oldest son*,* he tends to like to get out and be able to roam free*,* you know*,* and kind of run where there is no end in sight*,* you know. That’s a beautiful thing about being out in nature. You know*,* and being able to allow him to do that freely and*,* you know*,* not having any boundaries for him. I think that’s a beautiful thing. Watching him interact with wildlife*,* so being able to see the birds and name all of them and just being exposed to bugs and different creatures. It’s a fun experience watching him learn the world.*” (Parent).

Parents also emphasize the value in using nature to teach their children about autonomy as well as distinguishing danger from safety. It is important for parents to have the tools to help their children prepare for unpredictable situations.

“*Because as our children get older*,* mom and dad aren’t there to make every decision or hold their hand as they make every decision. And so they need to learn the pros and the cons of nature so that when they’re able to make their own decisions that they can think things through in that way and say*,* oh*,* no*,* I probably shouldn’t go near this bear. Because I know that the bear is dangerous*,* whereas if we didn’t talk about the negative con*,* you know*,* and you see a baby little cub walking around like*,* oh*,* I want to go pet it. Yes*,* it looks fuzzy and yes*,* it contributes to nature*,* but we don’t want to go near it.*’’ (Parent).

Parents feel that some choices involve more autonomy than others. Simultaneously, they perceive many choices along a spectrum of natural versus unnatural. Parents feel choices perceived as “more natural” are intuitive and responsive to biopsychosocial feedback that provides them more independence in personal and family-care contexts.

“*I do think it’s a good thing to discuss*,* because you should have a choice. I don’t think it should be forced into putting your child on a medication and worrying about their liver failing or the kidneys failing. So I think yeah you should have a choice. Do you want the natural stuff and just need a little more healthier and better*,* you know*,* or do you want the red prescription drug?”* (Parent).

Many parents feel an acute scarcity of nature dictated by temporal changes in the environment secondary to changes in weather, climate, human development, and their own life choices. In this context, parents and families feel a loss of control in raising their children and helping them build independence.

*“The development of the world and you hear about all these things that*,* you know*,* animals and things that are going extinct because of global warming and things like that. So I want her to appreciate the things that she sees*,* you know*,* every day and really absorb it because one day what if there aren’t these things? Well*,* what’s going to happen? Or if we were to live in a different state or a different town*,* like there would be different things and each would have to offer us than what they are here. And I would just want her to be able to appreciate what she has in that moment.”* (Parent).

### Activity suggestions

Taking the visit outside was commonly suggested by parents and clinicians. There was a perceived benefit to mental health and sense of wellbeing when taking a visit outdoors. There were many suggestions from parents and clinicians to “bring outside inside” with other developmentally appropriate activities that bring plants and other organic elements into the clinical space. Some clinicians suggested world building experiences with immersive language-centered or audiovisual activities. Some activities that were suggested have already been explored in a group medical visit setting and some were novel [1, 35]. [Appendix A includes documentation of GWCC-related activity suggestions from parents and clinicians.]

## Discussion

This study explored the integration of ecology-focused content into GWCC by examining the perspectives of parents and clinicians. Four primary domains emerged from the iterative process: the appropriateness of ecology-focused interventions in the GWCC clinical setting, the educational support desired by parents and clinicians, clinician commentary on perceptions and understanding of nature, and parents’ desire for independence and exploration. Our findings provide information on diverse perspectives regarding the acceptability, feasibility, and implementation strategies for ecology-focused GWCC adaptations.

Both GWCC parent-graduates and ecology-focused child clinicians felt that ecology-focused GWCC activities may be feasible to incorporate in the clinical setting. Similar to past research, clinicians recognize that there are wide disparities in access to economic resources as well as natural environments that feel safe and accessible [36, 37]. Clinicians felt strongly about ensuring ecology-focused GWCC redesign centers equitable healthcare delivery, which also was found in past research [31, 38]. While clinicians were worried about being inclusive to parents from diverse backgrounds in clinical care settings, GWCC parent-graduates already had frustrations about the status quo of individual WCC that they felt GWCC addressed [14, 20, 39]. Parent-graduates are supportive of WCC redesign and innovation as the status quo of WCC already brings many of its own challenges. The change in relationship between clinician and patient in group medical visits versus individual medical visits has been previously suggested as a significant mediator of outcomes in the perinatal and postnatal setting [10, 40]. There may be significant motivation from patients to better understand their clinicians’ opinions and perspectives on activities in their daily lives, be part of more low-stress environments, and feel more comfortable in the medical setting. An ecology focus may facilitate some of these changes and build on barrier-shifting that GWCC has already achieved.

Both parents and clinicians are interested in different forms of educational support around how to go out and do more ecology-focused activities. Clinicians were interested in bringing educational resources to their curricular redesign with topic specialists as well as coordination with other education-focused community entities. The desire for specialized support alongside community coordination speaks to the potential of problem-based or affinity-based groups in GWCC just as research has shown in GPNC [35, 41–43]. Clinicians and parents in GWCC may benefit from being connected to teaching kitchens, environmental educators, community gardens and/or transportation safety professionals that aid in facilitating one problem-based GWCC visit inside a visit curriculum that addresses many topics over multiple longitudinal visits.

Parents largely welcomed the idea of incorporating ecology-focused activities into GWCC, viewing it as a beneficial addition to clinical care. They expressed a need for more guidance on child safety and risk assessment when interacting with nature. Unintentional injury prevention counseling has been a staple of pediatric preventive care and has been standardized through the implementation of TIPP (The Injury Prevention Program), a program which was recently formally evaluated in a cluster-randomized trial and found to reduce parent-reported injuries [44, 45]. Surveys of pediatricians have revealed that 40% of children receive injury prevention counseling at a WCC. GWCC clinicians may want to consider how an ecology-focused GWCC approach can introduce environmental injury prevention counseling [46].

Clinicians, while supportive of this curricular alteration, recommended a personalized approach to accommodate diverse family backgrounds and emphasized the importance of leveraging local resources to design activities. Desires from clinicians to tailor GWCC as a place-based intervention raises the question of how to envision such a heterogeneous intervention at a scale which would encourage others to consider an ecology-focused GWCC model in their own communities. The RECETAS project to study nature-based social prescribing in the EU and GROWBABY research network for CenteringParenting® demonstrate how practices in different locations can share research infrastructure, outcomes, and protocols that make their models easier to translate to other local contexts [47, 48].

Conceptualizations of nature can be different among clinicians and parents based on their cultural backgrounds. These differences in understanding of nature may influence clinician curricular design and family responsiveness [49–51]. Previous research on conceptualizations of nature across languages, worldviews, and cultures has identified one way to categorize how nature is broadly understood by humans: (1) humans as part of nature, (2) humans as separate from nature, and (3) nature as experienced within a spiritual dimension [52]. This categorization may be a useful ontologic tool to understand different approaches clinicians may use when designing GWCC preventative counseling with ecology-focused curricula. Previous research has explored how perceptions, beliefs, and attitudes about topics as diverse as cigarette smoking, vaccination programs, and child health policy has shaped the counseling behaviors of pediatric clinicians [53–56]. Differences in conceptualizations of nature among parents and clinicians may also influence counseling behaviors of clinicians by invoking the existential experience. The existential experience has been previously understood as a sensation of understanding life and mortality on a time course that goes beyond the individual experience [57]. In the fields of palliative care, attending to the existential experience has been argued to be essential to providing whole-person care [58]. Bringing an ecology-focus into group well-child care may shift some well-child visit conversations from pragmatic, disease-focused, or guideline-directed towards a speculative, experiential, or philosophical direction. Clinicians may want to plan for facilitation in a way that creates space for the potential shared existential experience alongside the guideline-directed and practical advice focused activities and discussions.

### Limitations and strengths

Limitations of this study include the absence of clinician participants that had experience facilitating GWCC. Clinicians with such experience may offer a different perspective on the feasibility of ecology-focused adaptations to GWCC, considering competing priorities and resource constraints [59]. Another limitation is that the opinions of GWCC parent-graduates were obtained via a convenience sample. Parents who are more systematically sampled, with GWCC experience or not, may provide a different perspective on ecology-focused adaptations in GWCC, particularly if they were imagining a first experience with GWCC with a new child. The convenience sampling strategy used to select both clinician and parent participants may have led to selecting highly motivated candidates and more effort may be required to search for more generalizable perspectives on an ecology-focused approach. Among the strengths of this study is that this is the first study to provide comparative perspectives of clinicians and parents with experience in GWCC on GWCC redesign. Our qualitative approach allows the paper to engage in a nuanced discussion about integrating this ecology-focused approach in GWCC, highlighting diverse perspectives from parents and clinicians on topics pertinent to pediatric anticipatory guidance including physical activity, diet & nutrition, and the home environment.

## Conclusion

Overall, the study underscores the potential for ecology-focused GWCC to enhance pediatric anticipatory guidance by fostering connections between nature and child health. Parents and clinicians endorsed the importance of this approach, expressed unique preferences for participating in this model, and suggested a variety of feasible activities that may be appropriately replicated in the GWCC setting. Future research can incorporate the practical suggestions by parents and clinicians for reasonable ecology-focused GWCC activities into a structured curriculum that can be assessed in a pilot implementation trial that measures developmental outcomes and parent self-efficacy, social support, and emotional wellbeing. More importantly ecology-focused GWCC redesign can account for parent and clinician preferences including strong desires for integration of local resources, interest in risk-based counseling for “nature exposures,” and diverse relationships parents and clinicians may have with the perceived natural world. Complex parent and clinician preferences can be more deeply outlined in larger, more systematic qualitative studies that tailor curricular implementation to community needs.

## Electronic supplementary material

Below is the link to the electronic supplementary material.

Supplementary Material 1

Supplementary Material 2

Supplementary Material 3

Supplementary Material 4

Supplementary Material 5

## Data Availability

The interviews generated during the current study are not publicly available due to protecting the privacy of the participants of the study but are available from the corresponding author on reasonable request. A more expansive selection of supplementary material in Appendices A, B, and C provide additional quotes from participants.
