# Supplementary material for: Perspectives from parents and clinicians on an ecology-focused approach to a group well-child care
Source: BMC Prim Care. 2025 Feb 1;26:22. doi: 10.1186/s12875-025-02718-z (PMC11786538; doi:10.1186/s12875-025-02718-z)
Supplement: Supplementary file 1 — Supplementary Material 1 [file 12875_2025_2718_MOESM1_ESM.docx]

**Appendix A**

*Activity Suggestions Selected Quotes from Parents & Clinicians*

| Activity Suggestions | Quotes |
| --- | --- |
| Eating & Sensory | - Yeah, I know this isn't really the area we're talking about, but I mean from a nutritional perspective, I think at very young ages again, even like a one year old is really interested in picking leaves off a plan and eating them. And so, if you have like an herb pot that's safe for the child to pick leaves off and eat, that could be a great way to expand their palette and get them engaged in a plant, even if they can't like take care of it yet. I think, at older ages, you could probably like. I don't know. 5 or 6 year old, could probably use these tools in a much more educational way to learn more about like the rain cycle with plants. Or you know, greenhouse effect, or start to learn very basic things like that. Maybe learn a recipe to cook with that's super simple and alright. And even I mean a 2 or 3 year old-- safe like toddler kitchen tools that they could chop up leaves using (Clinician). - I mean, as a child gets older, I could think of if somebody were to bring in a fruit, right, if the pediatrician were to bring a fruit in and say, "hey, we're going to talk about strawberries today." And then the kids can kind of feel and touch the strawberries and feel the different seeds on the outside and kind of make it like a sensory activity. I could kind of see that happening as they're a little bit older and they're able to understand,"hey, when I hand you a strawberry, you don't just grab it and smush it in your hands. (Parent) - Even for little babies, I am always encouraging parents to always be talking to them and reading to them. I think that even though it’s not an activity that the child itself is doing. We can start reading little stories and talking to them about little things. I think that probably the earliest you are going to see a child be more engaged and more verbal to a level that makes sense is 15 months. 12 months is some kids are precocious enough. I would say the earliest would be 15 months. 18 months for sure. By 2 years the kids are ready to get their hands in and participate. I don’t think it’s ever too early. We can start reading very simple stories to them. Even just doing sensory things, feeling the different textures of leafy greens, playing with different foods. I think that’s completely possible for the younger ones. As they become more verbal and more mobile, they can start engaging themselves. (Clinician). - I think that it would help them understand, like, how different things are, but they can still be, you know, that strawberries and blueberries can be just as equally effective when you eat them as fruits, right? But the strawberry looks very different than the blueberry. Well, why is that? Well, they grow on different plants. They have different nutrients. They, yes, they're both fruits. Yes, they're both good for you. But this is a blueberry versus this is a strawberry and it can just help teach the kids the difference between different types of fruits, different types of vegetables, how they're able to grow, how we grow them, how we get our hands on them, and how the whole process of it going from a seed to a plant, could in watching something grow and nurture, I think that that could be beneficial as well. (Parent) |
| Going Outside | - So I think just taking a walk, and while they're doing that talking or pointing out things that are in the environment can be a great way. Don't forget above, clouds and sunshine and moon and stars, and those kinds of things depending on the time of day. So I would say, walking and talking may be the activity that may be most easy for a lot of people. And you don't need a lot, other than age appropriate levels. But exploring, whether it's like a scavenger hunt or journaling, or some more concrete activity that might work particularly for school age children like asking them to look for certain things (Clinician). - I wish I had the opportunity to give my kid more nature based play learning anything. I don't like my kid being inside all day I feel like she needs the sunshine. She needs to run around she needs the fresh air. She needs that opportunity to grow and see and experience more things than just toys and TV and crafts. (Parent) - But setting that aside, if one of our priority topics is physical activity, then we can tie that to going outside and spending time outside. And it's taking walks and like working towards opportunity to get the 60 min of fresh air and an activity. And then , tying into the Reach Out and Read book that may address that topic, and provide opportunities for kids to see it in the book, and then to be able to see it in nature. That’s my ideal world (Clinician). - I think for group well child care, teaching about the benefits of it could be helpful. Going out and having group outside so that parents can see and can be taught as well could be really good. They feel like we have so much man made stuff now. We have parks and stuff that I feel like sometimes we forget about the nature park. Like people want the comfort that they have and that it shows a different side. It's great for the kids to unwind, unplug and get away from it all. And teaching that and showing that it could be really good. Like just being outside, it might definitely be less structured and there would definitely be parents having to like help with the kids stuff. But I feel like there's places that it could be done that's relatively safe that they could teach that, they could show it and the kids would get the time to engage with other kids outside. They could be digging, they could be exploring like what's going on around them. (Parent) - But yeah, I think then, also thinking about the fact that we are in a city that has a fair number of natural resources and if in their free time, if they have any, are they able to get on public transit and go to a park? Or go to the local beaches, and you know, be at the ocean. Thinking about like ways to take advantage of those natural resources that are around us (Clinician). |
| Caretaking of Plants & Animals | - And I really try, I think when I've given talks, it's funny. I gave a talk at an environmental association inside a building where all the windows and everything was closed. And I said "this is exactly what we're talking about here." So I immediately open the windows, open the shades, tried to do things outside and people loved it. So getting people outside, if you can, even if the weather is not optimal, I think I gave one talk and it was going to be cold out or rainy. I can't remember which it was, but I said bring warm clothes, bring an umbrella. And so even then it's teaching them that you can go out. It doesn't have to be 75 degrees and sunny, you can go out in different conditions (Clinician) - I use it for learning experiences. I guess most recently we thought it would be interesting learning to plant seeds and watch them grow and kind of work on responsibility as much as that makes sense for how little he is. But find him most frequently asking to water his plants or kind of looking at them. So I think it's been fun watching him build those simple responsibilities and I guess kind of based on your definition, we also have him doing it where he feeds the dog and kind of learning how to take care of the dog and the fish in the house and learning those responsibilities that way, which he seems to thrive on and honestly reminds us more often if we're kind of getting overwhelmed with something within the day. That those tasks needs to be done, which I think is really, really cool for his age. (Parent) - It can be super simple. I talk to parents about just growing things indoors. Something that is super simple is taking green onions and after you use the green part, you can keep the white part with the little roots and you can put them in a jar and grow them (Clinician). - My older son has been learning that he wants to interact with every creature that exists. So we've been trying to teach him the nice ones and the not so nice ones. And I don't know how to teach him that these sting, even though they're cute, you know, so not to play with them. So I guess that's been one of the things that we've been trying to teach him, you know, this is something you need to learn that could hurt you or that might be dangerous to you. (Parent) - I mean at at the very least, you know, going for a walk. You know we used to hear about hugging a tree. In terms of the environmental movement. But actually hugging a tree or working in with plants can be very soothing centering activities. Watering plants. Playing in the dirt (Clinician). - hat exercise is planting seeds. I mentioned about anybody can recycle, reuse, repurpose a container.Plastic container that unfortunately we use so many of them.Cut them in half. Put some soil, and hopefully, it a good source of soil that not from the nearby highway. You get some soil and plant the seeds of whatever fruit or vegetable we used last week to cook and do that with your child. 2 year old 3-year old, do it, and tend, water it and take care of it and see it grow, and you can even compare the growth of the plant with the growth of your child, and talk to the child about how important it is to nourish and hydrate. So there's so many concepts that you can talk about with a child who's growing and developing, and that parents can themselves, with all the resources that they have with the smart phone look for information about how to grow a garden, how to talk to a child about nature and just being outside when you do that activity. Just on that time in nature, it's something that is powerful. Even kids living in heat islands where they don't have much canopy. Maybe at some point in the month, going out of the city and going into more rural area and doing this kind of activities (Clinician). - I mean, I work in a university town like you do. And so I could bring in some people who are actually doing work in this area that, you know, would be very interesting to hear from. So some scientists in the area, I mean, that would be helpful. And then resources. You know, I think the topics are such that we're talking about things that are very familiar to people. You know, bringing in animals has its pluses and minuses, you know, that can go awry. I don't think you need much in the way of resources. I think that, you know, if you're going to do something with gardening, and, you know, this is a very obvious instance where dirt is good, and it's nourishing, and, you know, it's necessary and whatnot. So that's, that's a good resource. Contact with different animals, I suppose would be interesting going to a petting zoo and whatnot talking about-- I was really disturbed. I went to give a talk in Sweden, and they had a really well developed petting zoo with all kinds of different barn animals and whatnot (Clinician) |
| Language & Audiovisual Activities | - I would pick something and I'll try to be more specific. But I'd pick an activity, or maybe 2 or 3 activities. And it can be around reading. Go to the Public library, find a book about nature. You can either browse the shelves, or you can go up to the reference desk and ask for some help, because, again, what's their experience using libraries? Spend time reading the book, try to connect it to something you might do outside. Connect that for your child. What's their response? Bring that back next time, you know. What is that? And let's all share. Same with technology like if I'm trying to show an affirmational element of technology, right? You know, download this free app and make it into a game with your child about how many different bird songs you can identify, whatever the case may be. But come back so that way, it's not just a I'm gonna give you a suggestion, and you go off into the ether and you do it or not. I'm not gonna know. Right? You know, I don't want people to feel like we're grading them on it, or that they're given an assignment date. "Have" to do, but trying to close the loop in some way, shape or form (Clinician). - this is where books are so amazing. It gives you this opportunity to travel into worlds that go beyond your immediate environment. And so, a simple example is like seeing a volcano, a picture of volcano in the book. Something that in the northeast USA, you can't walk down the street and see a volcano (Clinician). |
