# Supplementary material for: Perspectives from parents and clinicians on an ecology-focused approach to a group well-child care
Source: BMC Prim Care. 2025 Feb 1;26:22. doi: 10.1186/s12875-025-02718-z (PMC11786538; doi:10.1186/s12875-025-02718-z)
Supplement: Supplementary file 2 — Supplementary Material 2 [file 12875_2025_2718_MOESM2_ESM.docx]

**Appendix C**

*Commentary on Perceptions of Nature from Parents and Clinicians*

| **Perceptions of Nature** | **Quotes** |
| --- | --- |
| Nature & Temporality & Geography | - At the end, I just think about the children now, the people that aren't considered when laws, lawyers, and policymakers have their discussions, their decisions, and implementation of protocols. If they do or do not have the capacity, we have generations of kids that are exposed. We have a long way to go, and that includes water, and that includes air as well. In water you worry about phthalates. You worry about PFAS. And even you worry about lead. So many schools that have pipes. Leaded pipes. Their water has never been tested for in any given day. (Clinician) - The development of the world and you hear about all these things that, you know, animals and things that are going extinct because of global warming and things like that. So I want her to appreciate the things that she sees, you know, every day and really absorb it in because one day what if there aren't these? Well, what's going to happen? Or if we were to live in a different state or a different town, like there would be different things and each would have to offer us than what they are here. And I would just want her to be able to appreciate what she has in that moment. (Parent) - My power is surging on and off with the storm. Yeah. So, yeah, I mean, just, you know, things again that are out of your control. So, like, you know, there was that chemical explosion that happened over in West Virginia. You know, I feel terrible for people that have to go through that. I don't know what we would do if that's something like that happened here. I mean, you can't sell your house and move because there was a chemical explosion down the street. So things like that, I guess. OK.” (Parent) |
| Micro vs Macro Perspectives on Nature | - Other things or other parameters that I think about right away is there are a lot of theories about different chemicals that trees and shrubs give off that are beneficial to us in terms of our immune system and improving our immune system. And also if you look at the soil, having varied soil and playing in the soil, there are a lot of microbes in there that are healthy for our gut and our immune system. And so keep us healthier. (Clinician) - I mean, we love going for hikes right now. Also just loves digging in dirt. Looking for-- looking at different animals that exist. I think are really cool. And all the different sensory feelings that come out of everything that you can find in nature. Even just a variety of leaves versus how the tree feels and all that kind of stuff. (Parent) - Contact with animals in early life. And some of that is gonna build a healthy microbiome, but there may be other reasons why animal contacts are helpful. I mean, we can think of a lot of psychological reasons, but probably the animal proteins may actually influence immune development as well and other things that are associated with animals. And then being outside, because there's a fair amount of information of benefits of a biodiverse environment. It's not just the microbes that we get from animals, but it's being out with plants and nature. And some of those mechanisms are pretty well understood like vitamin D from sunlight and whatnot and some of them are just starting to be understood now, but there's lots of different signals. (Clinician) |
| Nature & Stewardship | - Ideally, nature is a part of everyone's daily life, right? (Clinician) - “It does influence my parenting style. If we have an ant in the house, it’s funny because my husband picks it up and throws it outside and he tells my son, it’s a living thing and it deserves to live like you. Don’t step on it. Don’t kill it. Just put it out. We try to teach him a lesson. Even if you’re outside, I try to teach him, “you’re outside, the ants are crawling so don’t step on the ant’s little house. Don’t touch the ants.” We try to teach him to respect the things that are outside and outdoors. (Parent) - I definitely think that our patients are aware of built environment and its influence on their child's health. I think the probably clearest example of that is related to respiratory concerns, and parents being aware of air pollution. Especially in the context of climate change, you know, living in near a factory or highway, or you know, the hot summer day with their smog. And then how that's associated with their kids breathing, and whether they're coughing or having an asthma flare. The'll say things like I don't think it's safe for them to go outside or play or like having trouble with this stuff. (Clinician) - “It's important to me because I want her to have everything that she possibly can. And I think that a lot of that has to do with understanding the way that her parents grew up and doing the things that we did when we were little and connecting with nature and understanding that we take from nature, but we also give to nature. And that without nature, the world wouldn't exist. So that it's just as important to, you know, spend time outside and really see the trees because one day there may not be certain things that there are outside today. (Parent) - I’m thinking always about inclusion and equity and accessibility of resources to promote education about nature, and how nature is the powerful resource that we overlook all the time. We're immersed in nature, we're part of nature. (Clinician) - Yeah, it's important. I'd say that it's grounding and it kind of puts us back in our place a lot as people, as humans. It kind of grounds us into "we're not the biggest and baddest necessarily all the time. (Parent) |
| Natural vs Unnatural | - Um, yeah, I really think that is true, because a lot of stuff like that I changed even like habits and different things. I used to be on high blood pressure medication I used to be on you allergy medicine different things and when I changed my diet, when I change, you know certain things on my own and I looked up certain stuff. I said “I can get off of this” and “I can eat this” and I can do this and you know “I can do this with my allergies.” So I think it's better and you know I'm healthier, instead of just pumping myself with medicine and same with my son (Parent) - Talking about the father. He can also learn about this is important. And then definitely having both parents involved. If it's a Latino family, you're going to have also, grandma, If they are not from United States or if they're migrants from other Latino countries...In Latino countries, we do have our support system families in a standard family. So everything that has to do with my kids when they're growing up. I had grandmas involved, and that's powerful, and they're going to tell you, "when I grew up, these things didn't exist, So I didn't use those products. That wasn't a problem. (Clinician) - I think learning the benefits of it could be really helpful and how to do things safely. Like a lot of people don't know what to do. Like, like go outside. Okay, we're now outside. What what can we do going on walks works. Like just digging holes. Like I don't feel like a lot of parents necessarily know what to do once they are outside and that we have a lot stricter now with clean and clean environments, especially since COVID. A lot of people are like hyper vigilant about keeping things clean that sometimes when we go outside, parents are kind of like don't get messy. Don't get dirty. And I wish that was talked more about like finding the balance between it all. (Parent) |
