# Supplementary material for: Perspectives from parents and clinicians on an ecology-focused approach to a group well-child care
Source: BMC Prim Care. 2025 Feb 1;26:22. doi: 10.1186/s12875-025-02718-z (PMC11786538; doi:10.1186/s12875-025-02718-z)
Supplement: Supplementary file 3 — Supplementary Material 3 [file 12875_2025_2718_MOESM3_ESM.docx]

**Appendix B**

*Comments on GWCC from GWCC Parent-Graduates*

| GWCC Suggestions | Quotes |
| --- | --- |
| Idea Generation | - “I mean I think having a medical professional along with everyone else's point of view in the group can be helpful for coming up with new ideas on how to engage with everybody in the natural environment. Because I know my career kind of puts me at thinking of those kind of things more often. But I always learn things from other people and you're like oh that's something I definitely didn't think of before that I think would help my child.” (Parent) - “I mean always like some general ideas on just like even kind of like a sheet of just ideas on how to get out and do things could be nice at the end. I mean, I wouldn't be opposed to us even going on little like adventures at the end and doing something outside together for the kids to just kind of interact with together, or bringing some sort of outside in. I don't know how that would look across all ages or what makes sense with it like not putting costs that aren't insurance related into it. But like, I don't know, almost having them get to do a project together of some sorts that involves more nature could be really fun. Yeah, I think mostly just like general ideas that could always be built upon as you learn from each age group could be fun just as a "here go out and try these.” (Parent) |
| Milestones | - “I think it would be really interesting or really kind of cool as like an aside or something to bring up in group. Because a lot of times group has done like six months or so. Once again, like little babies are done more regularly, but like, you know, right around when centering occurs kind of going through like whatever kind of things you could do out in nature with your kids, kind of having those kind of aspects and those different topics that you could kind of bring up as little like things.” (Parent) - “I think in Centering, we're pretty focused on the developmental milestones that are appropriate and the problems that people are having with raising their own kids. And so in the younger years, it tends to be my kid won't sleep or like my kid will only eat foods that are white and what do you guys do to help make sure that your kids get enough to eat? That's a better variety. So I don't feel like outdoor play has come up as a strong topic. I can't say that it hasn't been like mentioned, but I don't feel like there's been a huge emphasis on get your kids out of nature and let them see what they wanna do. I don't feel like that's come up a lot. I mean, I think it's possible that I've suggested to parents in the group discussions, my kids are better regulated when they're outdoors and they eat better that night, you know, that might have come up. Make sure your kids are active and that will help them have a better appetite and be a little bit more willing to try new thing.” (Parent) - “No. No. I mean, I think it would be nice if we could incorporate... Well, we're kind of limited, right? So we only are in there for about two hours. So they try to address all of our questions as much as possible. But I think that it would be nice if we could do a look ahead. So you know, at a four month visit what what to expect is going to happen between four and six months or six and nine months or nine months in a year. Like not only what the things the challenges that I'm looking at right now, but what am I going to look at in a month and go, what do I do? And I think that that would be beneficial, especially as a first time parent.” (Parent) |
| Staying on Topic | - “I mean, maybe just exploring the subject with us, having this conversation like we're having and then it's really hard to tell a toddler but our doctor always finds the right way to like, she always says, redirect or different things like that. So maybe kind of going through these examples of what we go through in our real life and again, kind of workarounds to, you know, make being outside safer and more fun for everybody.” (Parent) - “I don't know a situation when I feel like because she's used to not to give any information. So like all of our interactions have been get and get out of the doctor's office. I mean, Centering hasn't been. We sit there and we talk, but sometimes it can be a little all over the place. So I don't remember everything that's said, but I feel like that we haven't talked about it much with nature or anything to be able to can give an example” (Parent) |
| Changing the Space | - “Yeah, I absolutely do think that meeting outdoors would be awesome. It may be tricky to arrange, but if they were to get a pavilion, then they could be indoor/outdoor if there were rain or something. But they could meet outside, which would be super cool. They could, when they're talking about upcoming milestones or current milestones, they could use nature-based examples of those milestones. And that would just even subconsciously remind the parents of things that they can be doing outdoors with their kids.” (Parent) - “I think for group well child care, teaching about the benefits of it could be helpful. Going out and having group outside so that parents can see and can be taught as well could be really good. They feel like we have so much man made stuff now. We have parks and stuff that I feel like sometimes we forget about the nature park. Like people want the comfort that they have and that it shows a different side. It's great for the kids to unwind, unplug and get away from it all. And teaching that and showing that it could be really good. Like just being outside, it might definitely be less structured and there would definitely be parents having to like help with the kids stuff. But I feel like there's places that it could be done that's relatively safe that they could teach that, they could show it and the kids would get the time to engage with other kids outside. They could be digging, they could be exploring like what's going on around them.” (Parent) - “ I think for group well child care, teaching about the benefits of it could be helpful. Going out and having group outside so that parents can see and can be taught as well could be really good. They feel like we have so much man made stuff now. We have parks and stuff that I feel like sometimes we forget about the nature park. Like people want the comfort that they have and that it shows a different side. It's great for the kids to unwind, unplug and get away from it all. And teaching that and showing that it could be really good. Like just being outside, it might definitely be less structured and there would definitely be parents having to like help with the kids stuff. But I feel like there's places that it could be done that's relatively safe that they could teach that, they could show it and the kids would get the time to engage with other kids outside. They could be digging, they could be exploring like what's going on around them.” (Parent) |
| Benefits | - “Not right now. But again, like, as she gets older, how do you introduce ... at what point do you tell your kid to stop rolling around in dirt and putting it in their mouth? You know, and I'm sure that as she gets older, those conversations are going to kind of become more natural. But at this point in time, every concern that I've had has been a discussion in our Centering Parenting group that has kind of helped ease my fears as far as exposing my child to them or things of that nature.” (Parent) - “Oh, it's great. I love all of those people. It's always nice to kind of have an open environment to talk about any concerns or any issues that any specific family is having. And maybe you didn't have that question prior to when exploring that you realize, oh, wow, that's really helpful. You know, I wouldn't have thought to ask a question like that.” (Parent) - Well, when my daughter was a baby and we were all going through the teething stuff at different stages, we could all give each other advice. She got a little bit older in the table feeding and actually feeding themselves, talking about different foods and experiences and going out to a restaurant and how they do behavioral wise. I like being able to make that connection with other parents about similar things and unsimilar things in case I like having the knowledge of, oh, you went through that. et me remember in case it happens to me.” (Parent) - “I think that the best utilization of their time is being able to kind of see where the parents are and the parents' relation to the kids? Because a lot of times you can't see that as one-on-one in a doctor's office. I know my son specifically freaks out at the doctor's office. So they of course just see me on guard and very just vigilant about his care and well-being and things. And instead of saying me relax, okay, he is playing off with kids his own age, friends, and then kind of seeing parents are also in a little bit more of a psychologically, emotionally receptive frame of mind when their kids aren't screaming.” (Parent) |
| Who Comes to Group Well Child Care? | - “I think it can be incorporated because we have such a diversity in parents, a lot of people are from different places. So I'm pretty sure a lot of them incorporate natural stuff anyway, even like natural remedies. Sometimes I do like natural remedies. As far as medication, you know the honey and the tea and so I think it can be incorporated.” (Parent) - “I really don't think it will be hard to incorporate at all because right now I think when I went, it was a while ago a long while. I think people are ready to hear, you know, all these ideas, and I think they're open to it especially now after the COVID and different things that are going on.” (Parent) |
