# Supplementary material for: Perspectives from parents and clinicians on an ecology-focused approach to a group well-child care
Source: BMC Prim Care. 2025 Feb 1;26:22. doi: 10.1186/s12875-025-02718-z (PMC11786538; doi:10.1186/s12875-025-02718-z)

**Protocol for Literature Review for Domain Identification**

Primary Search: Using Google Scholar, search terms were inputted as follows: “nature” AND “pediatrics,” “environment” AND “pediatrics,” “ecology” AND “pediatrics,” “nature” AND “child health,” “environment” AND “child health,” “ecology” AND “child health”. The first 50 to 100 papers were reviewed by title to determine relevance to the ecology focus of the study as defined by experiences/concepts where human-nature interaction and its interconnected relationships was perceived to be important to parents and clinicians due to social and cultural relevance. Exclusion criteria were the use of the terms “nature,” “environment,” and “ecology” to describe sociological and medical phenomena in a structured way rather than describing the interconnected relationships between living and nonliving beings and its relationship to children’s health and healthcare.

Domain Identification: Papers that met the inclusion criteria on the initial search were initially screened for domain ideas. Domain ideas were iteratively generated from the read papers. The primary author (ITS) listed out phrases that may encompass a broad range of concepts described in the papers that were read.

Domain Hypothesis Testing: When the primary author felt that some of these phrases could be grouped together under a more general term, these terms were collapsed under a more general domain. These hypothesized domains were independently reviewed during this iterative process by co-authors RBJ and NH who provided feedback if certain topic areas did not seem to appear in this iterative search.

Iterative Secondary Search: After the primary search was conducted, potential domains were searched through Google Scholar alongside the search terms “pediatrics” or “child health” and the first 20 to 30 papers were reviewed for relevance. Papers were included in a secondary review if they were relevant to the search terms. These papers were reviewed to identify any new domains which covered areas that were not reasonably categorized in the existing list of domains. New domains went through the same iterative secondary search process after being identified.

Domain Finalization: The final list of domains was reviewed in a variety of ways. They were reviewed against the AAP Bright Futures anticipatory guidance to see if covered topics relevant to pediatric primary care by ITS. These domains were reviewed for any gaps by RBJ and NH for topic areas they have come across in their own personal and professional work that may be relevant to nature and children’s health. They were also reviewed against the relevant papers identified in the primary and secondary search for relevance based on whether topics covered in each paper could always fall under at least one or more of the generated final domains.


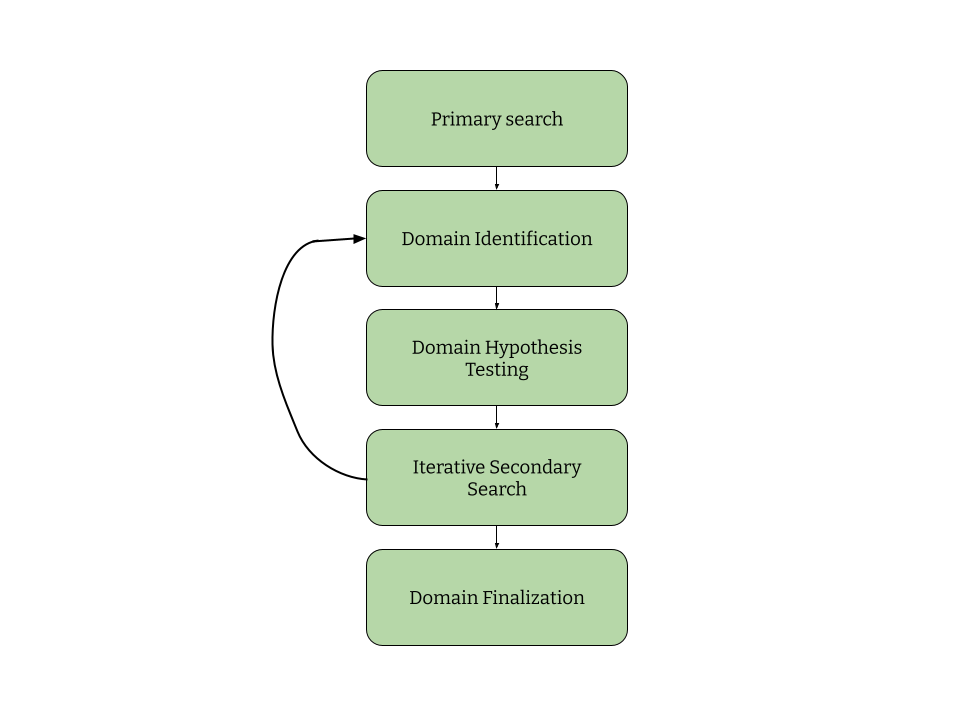

Supplement: Supplementary file 4 — Supplementary Material 4 [file 12875_2025_2718_MOESM4_ESM.docx]
