# Supplementary material for: Perspectives from parents and clinicians on an ecology-focused approach to a group well-child care
Source: BMC Prim Care. 2025 Feb 1;26:22. doi: 10.1186/s12875-025-02718-z (PMC11786538; doi:10.1186/s12875-025-02718-z)
Supplement: Supplementary file 5 — Supplementary Material 5 [file 12875_2025_2718_MOESM5_ESM.docx]

**Previous Group Well-Child Care Participant Semi-Structured Interview Guide**

**Introduction**

- “Good _____ .This is______ from Boston Medical Center Vital Village CRADLE Lab. I am _______ a research associate and will be facilitating the call today. Welcome and thank you for joining us for this interview.
- We are interested in your experience as a parent and the role of nature, your thoughts about nature and group well-child care, and your ideas about how to improve group well-child care.
- We encourage you to not refer to yourself or other people you may refer to by name during this interview.
- Before we get started, we will read off a brief information sheet about the study. [Read Brief Information Sheet for Previous Group Well Child Care Participants]
- What questions do you have about the statement that I have just read?
- Alright, let’s get started.

“First, we want to briefly touch on a definition for the environment and nature. We know this can mean a lot of things to a lot of different people so here is the interpretation we are using in our project. For this study, nature includes the physical world collectively, including plants, animals, the landscape, and other living or nonliving features and products of the earth. The environment includes national parks but it also includes city blocks, community gardens, and backyards. It includes houseplants and pets but it also includes the microbes in our soil, the bacteria in our gut, and the wildflowers growing between cracks in the pavement. We assume these definitions are incomplete and invite you to think within or beyond what we have described.

My first question is…”

1. What does nature mean to you? [Probes: What comes to mind when you think of nature? What counts? What doesn’t count?]
2. What types of experiences have you had with your child in nature? What comes to mind first? [Probe: what made that experience special? Did nature play a role?]
3. Is it important to you to spend time with your children with nature/ the environment? [Probe: Why or why not? If yes, where do you like to spend time]
4. Can you share an experience where nature/ the environment made you feel unsafe or was dangerous?
5. Is it important to talk about natural or environmental hazards (aspects of nature/ the environment that made you feel unsafe)? Why?
6. What are the challenges or barriers in avoiding natural or environmental hazards? [Probe: If yes, please describe]
7. How does nature/environment influence your parenting style?
   1. If not, why do you think it wasn’t as much of an influence?

**Transition**

- “Now we are going to shift the conversation to group well-child care and the intersection of pediatrics and nature. My next question is…”

1. How do you think the natural environment impacts child and family health? If so, can you share how it can positively impact child and family health? Can you share how it can negatively impact child and family health?
2. What conversations have you had with a medical provider about nature and child health? Where have they happened?
   1. Probe: [If quiet] Examples might be X X X - What experiences have been similar?
   2. Probe: Tell us more about that experience.
   3. Probe: Have others had similar experiences?
3. Did your pediatrician mention any of these connections? If so, can you describe how this was shared by your pediatrician?
4. Have you ever received guidance from your pediatrician about how to engage in the natural environment (with your child/family)?
   1. Probe: If yes, please share an example.

**Transition**

- “Now we will transition to some questions about the incorporation of nature and the environment into group well-child care .. My next question is…”

1. How would you describe your experience with group well-child care (also called Centering Parenting)? [Probe: Can you share an example or a story?]
   1. What worked well in your group well-child care experience and you would like to see continue? What would you like to see done differently?
2. Do you have ideas about how learning about the natural environment could be included in the group well-child care curriculum?
3. Where do you think it could be important (play, language, housing, poison, nutrition)?
4. What do you think pediatricians/healthcare providers should/could do with the additional time?
5. In an ideal world, what kinds of activities related to nature/environment would you have liked to do with your toddler?
6. What do you think are barriers or challenges to including this in group well-child care?
   1. (Probe : What strategies might be important to consider to account for these barriers and challenges?)

*Thank you for taking the time to do this interview. We really appreciate you sharing your insights with us. Would it be okay to send a REDCap survey to the email that we’ve been using to communicate? The survey collects some information in order for us to be able to mail you a $40 clincard.*

- *If they ask what information: name, email, phone number, mailing address, and birthday.*

*You will receive the Clincard in the mail in the next couple of weeks. Make sure to use your Clincard within the next six months, because if there is no activity on the card, there is a $5 monthly inactivity fee. When it arrives, email us in order for it to be activated. Would you like our email again?*

*Would you like to receive payment reminders or Clincard updates via text or email or both or neither?* [log in Access Clincard form]

*In REDCap, fill out name and email in the RA: Clincard Form/Update Tracker. Send the Event 1 Clincard Form to the participant if they want the $40 clincard. Set recurrences every 3 days for 5 times.*

**Ecology-Focused Child Clinician Interview Guide**

**Introduction**

- “Good _____ This is______ from the CRADLE Lab. I am _______ a Research Associate and will be facilitating the call today. Welcome and thank you for joining us for our interview. Today we hope to have a discussion about whether it’s a good idea to incorporate nature and the environment into group well-child care. We encourage you to not refer to yourself or other people by name during this interview. Before we get started, we will read off a brief information sheet about the study. [Read Brief Information Sheet for Ecology-Focused Child Clinicians] What questions do you have about the information that I have shared ?

**Transition**

“First, we want to briefly touch on a definition for the environment and nature. We know this can mean a lot of things to a lot of different people so here is the interpretation we are using in our project. For this study, nature includes the physical world collectively, including plants, animals, the landscape, and other living or nonliving features and products of the earth. The environment includes national parks but it also includes city blocks, community gardens, and backyards. It includes houseplants and pets but it also includes the microbes in our soil, the bacteria in our gut, and the wildflowers growing between cracks in the pavement. We assume these definitions are incomplete and invite you to think within or beyond what we have described.”

Next, we want to highlight some domains that you may have unique experience in with regards to the intersection of ecology and pediatrics The domains we have identified are: Nature and Affect/Emotional Wellbeing, Nature and Built Environment/ Toxins, Nature and Safe Exploration/Play/ Transportation, Nature and Nutrition/Diet, Nature and Microbes, Nature and Language/ Reading, and Nature and Technology. Please pick one or two domains that you feel you have unique expertise in that we can dive deeper into.

[After the environmental education/ health professional picks the domain(s), shift into the domain-specific questions that are listed below.]

**Nature and Affect/Emotional Wellbeing** (*Target Professional: nature and emotion child/family psychiatrist/ psychologist/ mental health professional)*

1. How does nature/ environment impact emotional wellbeing?
2. How can we use nature/ environmentally-centered activities to help parents develop healthy, loving relationships with their children?
   1. Probe: Where do these conversations seem to happen already?
   2. Probe: Other ideas might be [EXAMPLE] What else comes to mind?
   3. Standard Probe: Tell me more about that...
3. Themselves?
4. Each other?
5. Around what age should these activities be delivered (within 0 to 2)?
6. At what frequency should they be delivered?
7. Through what mode of delivery?

**Nature and Built Environment/ Toxins** (*Target Professional: housing focused pediatrician*)

1. How does nature/ environment influence discussions about housing/safe storage?
2. How can we use nature/ environmentally-centered activities to help parents have a conversation about housing/safe storage (free of lead, safe storage, poison prevention/response, full of light, community, etc.)?
   1. Probe: Where do these conversations seem to happen already?
   2. Probe: Other ideas might be [EXAMPLE]. What else comes to mind?
   3. Standard Probe: Tell me more about that...
3. How can we use nature/ environmentally-centered activities to help parents create their own sanctuary spaces at home?
   1. Probe: Where do these conversations seem to happen already?
   2. Probe: Other ideas might be [EXAMPLE]. What else comes to mind?
   3. Standard Probe: Tell me more about that...
4. Around what age should these activities be delivered (within 0 to 2)?
5. At what frequency should they be delivered?
6. Through what mode of delivery?

**Nature and Safe Exploration/Play/Transportation** (*Target Professional: greenspace pediatrician*)

1. How does nature/environment intersect with safe exploration/ play/ transportation?
2. How can we use nature/ environmentally-centered activities to help parents explore and generate new ideas on safe and engaging play?
   1. Probe: Where do these conversations seem to happen already?
   2. Probe: Other ideas might be [EXAMPLE]. What else comes to mind?
   3. Standard Probe: Tell me more about that...
3. How can we use nature/ environmentally-centered activities to help parents with diverse transportation availability think about how to get them and their children around safely and sustainably?
   1. Probe: Where do these conversations seem to happen already?
   2. Probe: Other ideas might be [EXAMPLE]. What else comes to mind?
   3. Standard Probe: Tell me more about that...
4. Around what age should these activities be delivered (within 0 to 2)?
5. At what frequency should they be delivered?
6. Through what mode of delivery?

**Nature and Nutrition/Diet** (Biodiverse, Plant-Centered Diets) (*Target Professional: lactation consultant, food security-focused pediatrician*)

1. How does nature/environment intersect with nutrition and diet?
2. How can we use nature/ environmentally-centered activities to help parents talk about and generate new ideas on healthy diet and nutrition?
   1. Probe: Where do these conversations seem to happen already?
   2. Probe: Other ideas might be [EXAMPLE]. What else comes to mind?
   3. Standard Probe: Tell me more about that...
3. How can we use nature/ environmentally-centered activities to help parents talk about breastfeeding?
   1. Probe: Where do these conversations seem to happen already?
   2. Probe: Other ideas might be [EXAMPLE]. What else comes to mind?
   3. Standard Probe: Tell me more about that...
4. How can we use nature/ environmentally-centered activities to help parents talk about diet transitions?
   1. Probe: Where do these conversations seem to happen already?
   2. Probe: Other ideas might be [EXAMPLE]. What else comes to mind?
   3. Standard Probe: Tell me more about that...
5. Around what age should these activities be delivered (within 0 to 2)?
6. At what frequency should they be delivered?
7. Through what mode of delivery?

**Nature and Microbes** (*Target Professional: pediatric infectious disease physician/ pediatrician epidemiologist*)

1. How does nature/ environment intersect with microbes in the context of pediatrics?
2. How can we use nature/ environmentally-centered activities to help parents learn more about microbes/ viruses, vaccines, and immunity?
   1. Probe: Where do these conversations seem to happen already?
   2. Probe: Other ideas might be [EXAMPLE]. What else comes to mind?
   3. Standard Probe: Tell me more about that...
3. How can we use nature/ environmentally-centered activities to help parents learn more about cleanliness and sickness in baby?
   1. Probe: Where do these conversations seem to happen already?
   2. Probe: Other ideas might be [EXAMPLE]. What else comes to mind?
   3. Standard Probe: Tell me more about that...
4. How can we use nature/ environmentally-centered activities to help parents learn more about waste and toilet training?
   1. Probe: Where do these conversations seem to happen already?
   2. Probe: Other ideas might be [EXAMPLE]. What else comes to mind?
   3. Standard Probe: Tell me more about that...
5. Around what age should these activities be delivered (within 0 to 2)?
6. At what frequency should they be delivered?
7. Through what mode of delivery?

**Nature and Language/ Reading** (*Target Professional: Reach Out & Read pediatrician, environmental educator, librarian*)

1. What is the connection between nature/environment and language/reading?
2. How can we use nature/ environmentally-centered books/songs/activities to help parents explore and generate new ideas on language and audiovisual growth?
   1. Probe: Where do these conversations seem to happen already?
   2. Probe: Other ideas might be [EXAMPLE]. What else comes to mind?
   3. Standard Probe: Tell me more about that...
3. Around what age should these activities be delivered (within 0 to 2)?
4. At what frequency should they be delivered?
5. Through what mode of delivery?

**Nature and Technology** (*Target Professional: screens/technology-focused pediatrician*)

1. In Pediatrics, how does nature/ environment intersect with technology? (such as apps or games or television shows )
2. How can we use nature/environmentally-centered activities to have discussions around the role of technology and screen-time in family life?
   1. Probe: Where do these conversations seem to happen already?
   2. Probe: Other ideas might be [EXAMPLE]. What else comes to mind?
   3. Standard Probe: Tell me more about that...
3. What kinds of activities can help us better understand the relationship between technology and nature?
4. Around what age should these activities be delivered (within 0 to 2)?
5. At what frequency should they be delivered?
6. Through what mode of delivery?

**Transition:**

- Now we want to dive into how these activities could fit within group well-child care.
  - What is group well-child care? The group well-child care model aims to educate mothers on healthy child development, effective parenting, and self-care while also providing a space for social support among patients. Parents with similarly-aged children meet together for a ninety minute to two-hour medical visit with their clinician and a peer provider (social worker, case manager, etc) where they get a health check-up and participate in facilitated discussion. Before we ask any questions, we will try to answer any questions you may have about the group well-child care model.
- Do you have any questions about group well-child care?”
- [Respond to any theoretical questions about group well-child care. Gauge their familiarity with the model and the flexibility of the curriculum.]

Transition

- “Alright, now that we have had an introduction to group well-child care, let’s start our questions. My first question is…”

1. Of the domains we have discussed, which area within these domains do you find most important to integrate into pediatric care? Probe: Can you share why this focus area is most important?
2. What are your ideas about how to structure a parent-child group learning setting *for this focus area?* Have you ever had conversations about *this focus area*? Where have they happened? Can you share more about the benefits and challenges about conversations involving this focus area?
3. Do you think this focus area could be incorporated into a group well-child care curriculum? Why or why not?
4. If you think this could be incorporated in a group well-child care curriculum what might be the best way to go about this?What principles should be followed and what should be avoided?
5. If you were to bring this back to your current practice, what might be your first steps in implementing this type of ecological pediatric education?
6. Where do you see other opportunities to introduce this type of ecological pediatric education into group settings with parents and children if time was not a barrier?

*Thank you for taking the time to do this interview. We really appreciate you sharing your insights with us. Would it be okay to send a REDCap survey to the email that we’ve been using to communicate? The survey collects some information in order for us to be able to mail you a $40 clincard.*

- *If they ask what information: name, email, phone number, mailing address, and birthday.*

*You will receive the Clincard in the mail in the next couple of weeks. Make sure to use your Clincard within the next six months, because if there is no activity on the card, there is a $5 monthly inactivity fee. When it arrives, email us in order for it to be activated. Would you like our email again?*

*Would you like to receive payment reminders or Clincard updates via text or email or both or neither?* [log in Access Clincard form]

*In REDCap, fill out name and email in the RA: Clincard Form/Update Tracker. Send the Event 1 Clincard Form to the participant if they want the $40 clincard. Set recurrences every 3 days for 5 times.*
